# The Transition from MODIS to VIIRS for Global Volcano Thermal Monitoring

**DOI:** 10.3390/s22051713

**Published:** 2022-02-22

**Authors:** Adele Campus, Marco Laiolo, Francesco Massimetti, Diego Coppola

**Affiliations:** 1 Dipartimento di Scienze della Terra, Università degli Studi di Torino, V. Valperga Caluso 35, 10125 Torino, Italy; adele.campus@unito.it (A.C.); marco.laiolo@unito.it (M.L.); francesco.massimetti@unito.it (F.M.); 2 NATRISK: Centro Interdipartimentale sui Rischi Naturali in Ambiente Montano e Collinare, Università di Torino, Largo Paolo Braccini, 2, 10045 Grugliasco, Italy

**Keywords:** VIIRS, MODIS, MIROVA, FIRMS, Volcanic Radiative Power, thermal remote sensing, volcano monitoring

## Abstract

The Moderate Resolution Imaging Spectroradiometer (MODIS) is one of the most-used sensors for monitoring volcanoes and has been providing time series of Volcanic Radiative Power (VRP) on a global scale for two decades now. In this work, we analyzed the data provided by the Visible Infrared Imaging Radiometer Suite (VIIRS) by using the Middle Infrared Observation of Volcanic Activity (MIROVA) algorithm, originally developed to analyze MODIS data. The resulting VRP is compared with both the MIROVA_MODIS_ data as well as with the Fire Radiative Power (FRP), distributed by the Fire Information for Resource Management System (FIRMS). The analysis on 9 active volcanoes reveals that VIIRS data analyzed with the MIROVA algorithm allows detecting ~60% more alerts than MODIS, due to a greater number of overpasses (+30%) and improved quality of VIIRS radiance data. Furthermore, the comparison with the nighttime FIRMS database indicates greater effectiveness of the MIROVA algorithm in detecting low-intensity (<10 MW) thermal anomalies (up to 90% more alerts than FIRMS). These results confirm the great potential of VIIRS to complement, replace and improve MODIS capabilities for global volcano thermal monitoring, because of the future end of Terra and Aqua Earth-observing satellite mission of National Aeronautics and Space Administration’s (NASA).

## 1. Introduction

Volcanic activity causes the variation of numerous geophysical and geochemical parameters that characterize the state of a volcano. Among these, the satellite-derived thermal flux is increasingly used to detect signs of unrest [1,2,3,4] or to follow the evolution of an eruption once the activity has started [5,6,7].

The first applications of this discipline date back to the 60s and 70s through the analysis of the High-Resolution Infrared Radiometer (HRIR), a sensor mounted on NASA’s meteorological satellites Nimbus-1 and Nimbus-2 [8]. Later in the mid-1980s, several studies demonstrated how the data provided by the Thematic Mapper (TM) of the Landsat missions [9,10] and by the Advanced Very High-Resolution Radiometer (AVHRR) of the Tiros missions [11] can be successfully applied for studying thermal emissions; these sensors have been used even recently, for example, to quantify thermal budgets of eruptions [12] and to build thermal satellite monitoring tools [13,14,15]. The development of algorithms capable of detecting and monitoring the different types of activities allowed to expand this field of study [16], also thanks to the availability of thermal data acquired from sensors such as the Along Track Scanning Radiometer (ATSR) and the Advanced Baseline Imager (ABI) mounted on the Geostationary Operational Environmental Satellite (GOES) [9,11,17,18].

A decisive turning point takes place starting from 2000 with the beginning of NASA Earth Observing System (EOS) missions. The Advanced Spaceborne Thermal Emission and Reflection Radiometer (ASTER), launched on the NASA’s Terra satellite on 18 December 1999 was the first orbital high spatial resolution multispectral TIR instrument with a notable focus on volcanology [19]. At the same time, the launch of the Moderate Resolution Imaging Spectroradiometer (MODIS) onboard of Terra (1999) and Aqua (2002) satellites, allowed the creation of the first automatic global volcanic monitoring system: MODVOLC [20,21]. Thanks to the availability of open data policies and increasing accessibility to IT tools, other volcanic hot-spot detections systems were developed, each based on diverse spectral, spatial or temporal criteria applied to different satellite imagery. All these approaches show some advantages and limits [22], and can be classified into four categories: fixed threshold (e.g., MODVOLC; [20]), contextual (e.g., MODLEN; [23] and VAST; [24,25]), temporal detection methods (e.g., RAT-RST system; [13]) and hybrid algorithms (e.g., OKMOK; [26]; MYVOLC; [27]). In this panorama, a new hybrid algorithm named MIROVA (Middle Infrared Observation of Volcanic Activity) has been developed by [28] to analyse infrared images acquired by MODIS in near-real-time (NRT). Although other volcano-dedicated algorithms have been developed for different sensors [5,16,21,22], MODVOLC and MIROVA are probably the most used for near-real-time thermal monitoring, both working on a global (or nearly global) scale using MODIS [7,29].

The potentialities of this sensor rely upon (i) the moderate spatial resolution (~1 km); (ii) the moderate temporal resolution (~4 images per day); (iii) the presence of a “fire-channel” (low-gain Middle Infrared (MIR) band: ~4 µm). Notably, both MODVOLC and MIROVA provide data in terms of Volcanic Radiative Power (VRP, in Watt), a measurement of the radiant power released by high-temperature volcanic features (see [20] and [30] for details). The VRP was intentionally inherited from the better-known Fire Radiative Power (FRP; see [31]) and shares the same approach for calculating the heat emission of sub-pixel hot-spots (the so-called MIR method; [31]). The VRP allows quantifying the radiant power over more than 5 orders of magnitude, thus covering a wide spectrum of recent eruptive activity on Earth [29,32,33] and allowing the first quantification of global radiant flux from Earth’s subaerially erupting volcanoes [32,33]. The usefulness of VRP has emerged especially during recent effusive crises [7] since this parameter allows the calculation of the lava effusion rates, one of the parameters that most controls the length and evolution of the lava flows [34]. Also, the short- to mid-term analysis of VRP trends has proved to be effective in tracking the sudden changes in volcanic activity [35,36,37], in recognizing the end of eruptions [38,39], or in revealing communications between volcanoes [40]. Indeed, the decade-long analysis of VRP time series at persistently active volcanoes (e.g., open vent, lava lakes, lava domes) constitutes a solid basis for identifying background, threshold, and anomaly values that may help to recognize variations in the long-term volcanic output [12] and detect unrest evidence [7]. Due to these robust features, the VRP has been recommended by the 2017–2027 Decadal Survey-Thriving on our Changing Planet, as a targeted parameter within the “Surface Biology and Geology Designated Observable” [41,42].

Although both MODIS sensors are still in orbit, well beyond their designated lifetime of 6 years [43], data acquisition is only guaranteed until 2026 [29,43,44]. The imminent disposal of Terra and Aqua platforms makes it necessary to use new satellites and sensors to continue measuring the VRP for the next decades, in continuity with the legacy left by the MODIS-based volcanic hot-spot detection systems. 

To ensure continuity in the acquisition of critical environmental observations the last decade sees the beginning of a new generation of environmental satellite monitoring missions, operated by the National Oceanic and Atmospheric Administration (NOAA) and NASA. These missions are operated by the Joint Polar Satellite System (JPSS) and since 2011, they have launched two satellites (Suomi-NPP and NOAA-20/JPSS-1) equipped with the Visible Infrared Imaging Radiometer Suite (VIIRS) [45]. VIIRS is a multispectral imaging radiometer with temporal, spatial, and spectral resolutions similar to MODIS thus representing one of the most valid candidates to continue the analyses of volcanic thermal flux on a global scale [46]. 

VIIRS applications for detecting and studying global wildfire activity and its impacts are well-known and validated, also by merging data from different satellites see [47]. However, in the last years, several authors use VIIRS-derived Fire Radiative Power (FRP) data from freely-downloadable Fire Information for Resource Management System (FIRMS; [48]) to study volcanic eruptions and activity [49,50,51]. However, there is still no work that has validated the FIRMS data for volcanological applications. Recently, few authors worked on the development of algorithms and systems dedicated to the thermal monitoring application of volcanic activity by using VIIRS data [52,53,54], but none of them is either operative on a global scale yet.

In this work, we test the exportability of the MIROVA algorithm, currently ingesting NRT MODIS images [55], to VIIRS images. The nighttime VIIRS data acquired during 2012–2020 at two persistent active volcanoes, Láscar (Chile) and Erta Ale (Ethiopia), were processed using the MIROVA algorithm to build time series of VRP (here called VRP_VIIRS_). This dataset was then compared with that obtained by the same algorithm applied to the MODIS data (VRP_MODIS_) as well as with that provided by the FIRMS algorithm [56] (this last resulting in time-series of Fire Radiative Power; FRP_VIIRS_). The three datasets are then analysed in terms of the number of alerts, the statistical distribution of VRP/FRP, and the cumulative thermal energies. 

**Figure 1:**
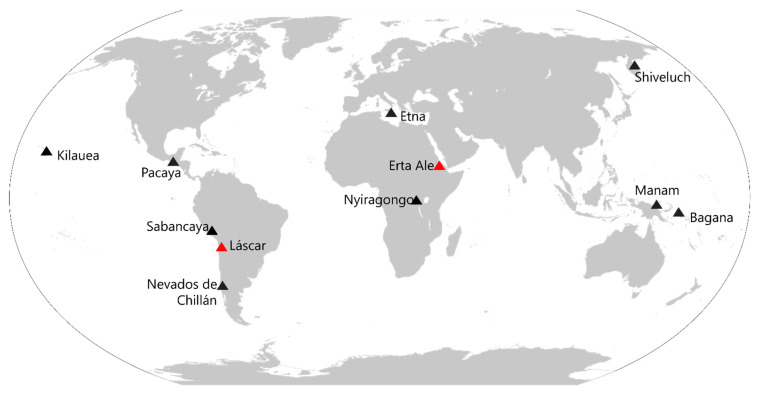
Geographic locations of volcanoes investigated in this paper. Red triangles indicate the two case studies (Láscar and Erta Ale volcanoes) for which we compared 9 years of nightime data using MIROVA_VIIRS_, MIROVA_MODIS,_ and FIRMS_VIIRS_ algorithms (see Section 4); black triangles represent the volcanoes for which we compare the MIROVA_VIIRS_ and MIROVA_MODIS_ algorithm to test the full potentiality (nighttime and daytime) of VIIRS for global thermal monitoring (see Section 5).

Finally, we expand our analysis by testing the MIROVA_VIIRS_ algorithm on a temporally limited dataset (1 year: 2021) which includes both data (day and night) acquired by the two VIIRS sensors currently in orbit (see Section 3.1). This test is performed over on 9 volcanoes characterized by very different types of activity (e.g., lava flows, lava lakes, lava domes, open vent activities) and located in very different environmental contexts (Figure 1). These preliminary results, provide a solid basis to permit the continuity of MIROVA and others hot-spot detection systems through the analysis of VIIRS data toward a global thermal volcano monitoring for the next decade(s).

## 2. Case Studies

The thermal activity investigated in this work is principally those retrieved on Láscar and Erta Ale volcanoes (Figure 1). We choose these two targets for an intercomparison of the three datasets (MIROVA_VIIRS_, MIROVA_MODIS_, FIRMS_VIIRS_) due to the following motivation:

1. both volcanoes were in persistent activity for the entire duration of the analyzed period (2012–2020). This makes thermal datasets large enough to allow a robust correlation (>3000 images analyzed at each volcano);
2. both volcanoes are located in arid areas with low cloud fraction, which favors the high alert detection frequency, and reduces the noise in radiative power time series;
3. both volcanoes are characterized by evident fluctuations of heat flux during the considered period. This feature allows to evaluate how the VRP/FRP responds to sudden variations of the heat flux over several orders of magnitude;
4. the two volcanoes are characterized by different kinds of activity (lava lake and basaltic lava flow for Erta Ale, and persistent fumarolic degassing and explosive activity for Láscar); thus we may evaluate the algorithm’s performance on volcanic thermal sources having different intensity and temperature distributions.

In the next paragraphs, we give an overview of the geological background and recent activity of the investigated volcanoes.

### 2.1. Láscar

**Figure 2:**
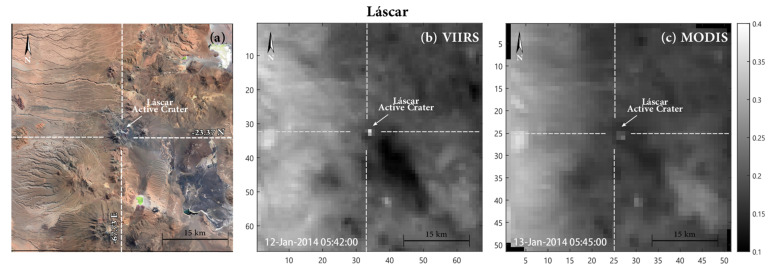
(**a**) Location of Láscar volcano (credits: Google Earth^®^); (**b,c**) examples of VIIRS (**b**) and MODIS (**c**) images acquired over Láscar. The images represent middle infrared (MIR) radiance data (band M13 and band 21/22, for VIIRS and MODIS, respectively) resampled and centered on the volcano summit at the original resolution of 750 m (for VIIRS) and 1000 m (for MODIS). Note how the different resolution allows a better definition of thermal anomalies. The bright pixels identify the thermal anomaly detected by the two sensors and related to the hot volcanic features present at the time of the image acquisitions.

Láscar (23.37° S–67.73° W) is a 5592 m high andesitic-dacitic stratovolcano located in the northern Chilean Andes (Figure 2) and it’s the most active volcano of the Andean Central Volcanic Zone (ACVZ) [9]. The ACVZ is formed by the subduction of the Nazca plate under the South American plate in a back-arc context [57].

The edifice of Láscar consists of two overlapping cones and currently hosts three summit craters that partially overlap. The westernmost of these craters is the one currently active, also known as “Active Crater” [58,59,60]. In the last century, this volcano has been characterized by cyclic lava dome extrusions and collapses, accompanied by Vulcanian to Plinian eruptions [58,61]. The last major eruption (VEI 4) occurred between January and August 1993 and was characterized by a series of phreatic to Plinian explosions that produced columns up to 25 km high and more than 0.1 km^3^ of ash [62]. Since then, Láscar had experienced 10 minor eruptions (VEI 2-3), the most recent in 2013 and 2015 [60,63,64]. The activity of the last decade shows a thermal pattern defining three yearly-long cycles having a similar trend. These cycles have been described by [65] and partially observed in dedicated periodic reports [66]. 

Because of its geographical conditions, the activity of Láscar volcano has been investigated mainly using satellite remote sensing data [9,10,63,67,68,69,70]. According to these authors, the source of thermal emission is located mainly at the bottom of the Active Crater (800 m wide, 400 m deep) where extensive fumarolic areas (having average temperature estimated between 300 °C [59,71,72] and 600 °C [73]) have been observed, eventually associated to the presence of permeable andesitic lava dome(s).

### 2.2. Erta Ale

**Figure 3:**
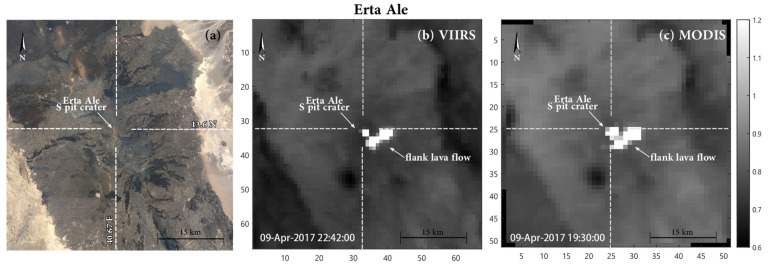
(**a**) Location of Erta Ale volcano (credits: Google Earth^®^); (**b**,**c**) examples of VIIRS (**b**) and MODIS (**c**) images acquired over Erta Ale. The images represent MIR radiance data (band M13 and band 21/22, for VIIRS and MODIS, respectively) resampled and centered on the volcano summit at the original resolution of 750 m (for VIIRS) and 1000 m (for MODIS).

Erta Ale (13.6° N–40.67° E) is the most active volcano in Ethiopia and one of the most active in Africa (Figure 3). This basaltic shield volcano lies on the about 120 km- NNW elongated Erta ‘Ale spreading segment within the Afar depression region, which represents the triple junctions between Nubian, Somalian and Arabian plane plate [74,75]. The 600 m-high edifice is cut by a 0.9 × 1.6 km elliptical summit caldera which hosts two pit craters [76,77]. For almost a century one or two lava lakes have been observed within these pit-crater(s), which made Erta Ale the volcano hosting the longest-lived lava lake(s) on Earth [76,78,79]. However, in January 2017, massive overflows from the lava lake inside the south pit-crater were followed by the first observed fissure eruption, who took place outside the caldera, along the southeast rift zone (Figure 3b,c). The effusive activity persisted until February-March 2020 and produced a large lava field covering ~28 km^2^ (data updated June 2019) [77,80,81,82]. This effusive phase has been investigated using various sensors (i.e., MSI Sentinel-2 and OLI Landsat-8), to evaluate new algorithms and new multi-sensor approaches for monitoring volcanic activity (cf with [83,84,85]). On the whole, all the authors agree in associating the persistent thermal emissions recorded until 2017 to the lava lake(s) within the pit crater(s) and/or to the occurrence of episodic overflows [80,86,87,88]. On the other hand, the highest thermal anomalies detected between January 2017 and March 2020 are related to the emplacement of the lava flows that occurred during the exceptional flank eruption. Since March 2020, several satellite monitoring systems identified discontinuous thermal activity sourced a new from the two pit craters [82].

## 3. Materials and Methods

### 3.1. Sensor and Products 

The Visible Infrared Imaging Radiometer Suite (VIIRS) is a whiskbroom scanning radiometer mounted on two platforms: the Suomi National Polar-Orbiting Partnership’s (SNPP) and the Joint Polar Satellite System’s (JPSS) JPSS-1 (NOAA-20 or N20) orbiting since October 2011 and November 2017, respectively [89,90]. The Suomi-NPP mission is a “bridge” mission born to continue NASA’s Earth Observing System (EOS) missions (which included Terra and Aqua satellites). VIIRS installation’s key objective is to guarantee a continuous acquisition of the parameters acquired by MODIS. Both satellites (Suomi-NPP and JPSS-1) allow full daily coverage of Earth thanks to their polar orbit, at a nominal altitude of 824 km [45,91]. VIIRS field-of-view (FOV) is 112.56° (13° higher than MODIS) in the cross-track direction and its swath width is 3060 km. Different from MODIS, the onboard acquisition scheme of VIIRS is characterized by an aggregation function that reduces pixel footprint growth across the scan by aggregating two or three raw samples. This function increases the pixel resolution at the edge of the images (Table 1) and extends the possibility of high-quality acquisitions over a higher satellite zenith angle [45].

VIIRS acquires in 22 spectral bands covering the EM spectrum between 0.412 μm and 12.01 μm, including 16 moderate-resolution bands (M-bands), with spatial resolution of 750 m, 5 imaging resolution bands (I-bands), with a spatial resolution of 375 m and one panchromatic Day-Night Band (DNB), with a 750 m spatial resolution throughout the scan. 11 of the 16 M-bands are Reflective Solar Bands (RSBs), 5 are Thermal Emission Bands (TEBs), while the I-bands include 3 RSBs and 2 TEBs [45]. 

In this work we used M13 and M15 M-bands, covering respectively the MIR (3.973–4.128 μm) and TIR (10.263–11.263 μm) portion of the spectrum, with a 750 m spatial resolution, which represent the corresponding MODIS bands 21/22 (MIR) and 31 (TIR), respectively. Table 1 shows the main characteristics of the VIIRS and MODIS sensors [92,93]. 

For this work, we used Suomi-NPP VIIRS Level 1B products which include the radiances data product (VNPMOD02-Moderate Resolution 6-Min L1B Swath 750 m) and the geolocation data product (VNPMOD03-Moderate Resolution Terrain Corrected Geolocation 6-Min L1 Swath 750 m Light V001). These two products are distributed by NASA’s Level-1 and Atmosphere Archive & Distribution System–Distributed Active Archive Center (LAADS-DAAC) [94], in netCDF4/HDF5 format files with sizes of ~200 MB and ~55 MB, respectively. We elaborated only the nigh time data acquired by Suomi-NPP from 19th January 2012 to 31st December 2020, which consists of a bulk dataset of about 0.5 Tb per volcano. To make the comparison with MODIS as homogeneous as possible we only used MODIS-Aqua nighttime acquisitions elaborated by MIROVA over the same time window.

### 3.2. MIROVA Algorithm and Calculation of Volcanic Radiative Power (VRP)

The Middle InfraRed Observation of Volcanic Activity (MIROVA) is a near-real-time monitoring system based on the processing of MODIS InfraRed data [28]. The system operates in near-real-time (NRT) for a list of ~220 active volcanoes and provides VRP time series used for monitoring ongoing volcanic activity [7]. The algorithm elaborates satellite images using spectral and spatial filters in search for high-temperature thermal anomalies, sourced by volcanic features. The NRT processing chain is made of 4 successive steps: (i) download; (ii) resampling; (iii) hot-spot detection and (iv) calculation of the VRP. As described below, steps 1 to 4 have been slightly modified from [28] and adapted to VIIRS due to its spatial, geometric, and spectral differences with MODIS (Table 1). 

We downloaded VIIRS L1B granules from the LAADS-DAAC system [94] and we extracted MIR (band M13) and TIR (band M15) radiances and the geolocation data related to our target volcanoes. Resampling is performed in a UTM 51 × 51 km grid, centered on the volcano summit (consistent with MODIS_MIROVA_ images) by keeping the nominal resolution of 750 m. This results in matrices of 67 × 67 pixels rather than 51 × 51 pixels obtained from MODIS (Figure 2 and Figure 3). 

The hot-spot detection algorithm is the same used for MODIS and based on spectral indices and statistical thresholds that automatically identify and localize pixels containing high-temperature thermal anomalies (see [28] for details). Once the hot-spot-contaminated pixels have been detected, we calculate the VRP by adapting the MIR method [31] to the VIIRS features:

(1) $$\mathrm{VRP}=\Delta L_{MIR}\cdot 1.97\times 10^{7}\cdot A_{pix}$$

where $A_{pix}$ is the pixel surface in km^2^ (equal to 0.5625 for VIIRS M-bands). The constant $1.97\times 10^{7}$ represents the best-fit wavelength-specific coefficient that takes into account the relationship between the Stefan-Boltzmann law and Plank’s Radiation law for a range of temperature between 600 and 1600 K [31,47]. In (1), the parameter $\Delta L_{MIR}$ represents the excess of MIR radiance, calculated as:

(2) $$\Delta L_{MIR}=L_{MIRhot}-L_{MIRbk}$$

where $L_{MIRhot}$ is the radiance of the alerted pixel/s and $L_{MIRbk}$ is the background radiance, calculated as the arithmetic mean of pixels surrounding the active one/s [28,30,47]. The VRP is calculated in Watts (W) and represents a combined measurement of the area of the volcanic emitter having an effective radiating temperature higher than 600 K [28].

### 3.3. FIRMS Database and Fire Radiative Power (FRP_FIRMS_)

The two volcanoes were analyzed by using the thermal anomalies provided by the FIRMS system over the same regions and time windows. In particular, we download the Suomi-NPP alerts, detected by the new VIIRS 375 m active fire detection algorithm [95], which combine the two MIR bands (at 750 m and 375 m) available on the VIIRS sensors. These data are archived since January 2012 and downloadable at https://firms.modaps.eosdis.nasa.gov/ (accessed on 16 December 2021), through temporal and spatial queries. 

The downloaded data consists of a single file (.csv) which includes the Fire Radiative Power (FRP_VIIRS_) values for each hot-spot contaminated pixel alerted in the period and the region of interest. Therefore, to be consistent with the VRP measured by the MIROVA system, for each satellite overpasses we aggregated the FRP of individual pixels to obtain the total FRP_FIRMS_ value for that image. It should be emphasized that the hot-spot detection algorithm underlying the FIRMS_VIIRS_ system [95] is different from MIROVA, this last being expressly developed to work on volcanic areas. However, the method of calculating the FRP_VIIRS_ is essentially the same as that used to calculate the VRP_VIIRS_, both based on the MIR method described by Equation (1).

### 3.4. Uncertainties and Limits 

The estimation of VRP through the MIROVA algorithm, or FRP through the FIRMS algorithm, is subject to several limitations that condition their direct application for volcano monitoring. 

First of all, there is a standard error of ±30% associated with the estimation of the radiant power through the MIR method [31]. This error affects the estimates of VRP/FRP, although the coefficients used in Equation (1) vary according to the sensor used (M13 at 750 m for VIIRS and B21/22 at 1000 m for MODIS; Table 1). Additional uncertainty in the estimation of the VRP/FRP may derive from the method used to detect the hot pixels (i.e., the hot-spot detection algorithm) as well as in the estimation of the method to estimate background radiance values (Equation (2)).

Several external factors may also contribute to increasing the uncertainty of the VRP. Indeed, the presence of meteorological and volcanic clouds, the satellite viewing angle, while false alerts, fires, or anthropogenic heat sources, may also introduce noise in the time series and a higher uncertainty in the single-point interpretation. For this reason, during an eruptive crisis the data provided by MIROVA, as well as that eventually obtained from the FIRMS, must always be evaluated and interpreted by an end-user that takes into account the real acquisition conditions of each hot spot [7]. However, for a comparison between the time series, in this work, we have left the datasets “as they are”, that is without applying image inspections or filters that discard cloudy scenes or scenes acquired in unfavorable geometric conditions (e.g., high satellite zeniths). Actually, under these conditions, we test the potential efficiency of the algorithm in NRT applications where such supervision is not applied.

## 4. Results

In this chapter, we present the results obtained at Láscar and Erta Ale volcanoes by comparing the three datasets (VRP_VIIRS_, VRP_MODIS,_ and FRP_FIRMS_, all in Watt) as obtained by the MIROVA and FIRMS algorithms.

### 4.1. Láscar

**Figure 4:**
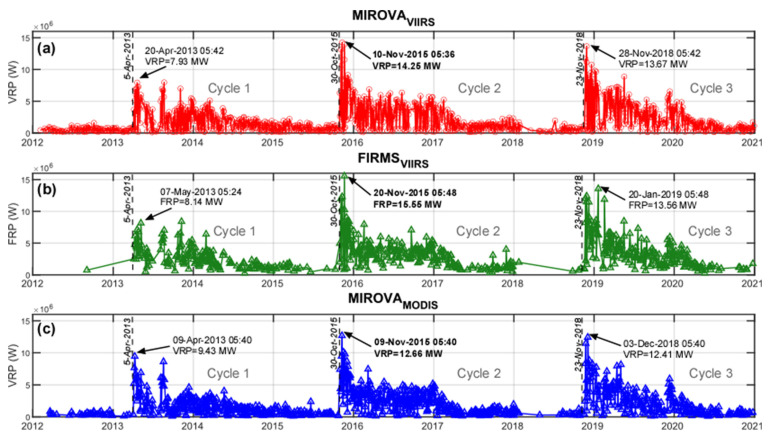
Time series of Láscar volcano. The panels show VRP calculated respectively from (**a**) MIROVA_VIIRS_ (red circles), (**b**) FIRMS_VIIRS_ (green triangles), and (**c**) MIROVA_MODIS_ (blue triangles). The black dashed lines represent the date on which a sudden change in the VRP, associated with the beginning of a cycle, was detected by the three datasets. The arrows highlight the peaking VRP recorded during the following days. Note that, even if the high thermal phases reach their maximum detections on a different day, the inferred onset is detected by all systems within a time window of 24 h, with few differences in magnitude, probably due to instrumental and algorithm-related features.

The Láscar time series derived by both sensors and algorithms (Figure 4) are characterized by nearly continuous detection of thermal anomalies resulting in a number of alerts (N_alert_) equal to 1221 for MIROVA_VIIRS_, 875 for MIROVA_MODIS,_ and 627 for FIRMS_VIIRS_ (Table 2). These data suggest that over Láscar volcano the MIROVA_VIIRS_ algorithm detects 40% more alerts than MIROVA_MODIS_ and 95% more than FIRMS_VIIRS_. By taking into account the number of MODIS and VIIRS overpasses (N_pass_) the above data translates into a frequency of alert detection (f% = N_alert_/N_pass_) of 30%, 28%, and 15% for MIROVA_VIIRS_, MIROVA_MODIS,_ and FIRMS_VIIRS_, respectively (see Table 2). These percentages suggest that the higher number of MIROVA_VIIRS_ alerts is not simply attributable to the higher number of VIIRS overpasses, but also to a greater sensitivity of VIIRS data to the presence of small thermal anomalies.

The trends defined by all the time series (Figure 4a–c) are very coherent and show at least three main eruptive cycles characterized by a sudden peaking phase (with VRP ≥ 8 MW), followed by a waning phase lasting several years (with VRP decreasing below 1 MW). These three cycles are part of a major phase of Láscar activity (identified as “Phase 4” in [65]) which began in April 2013 in correspondence with our first cycle. Based on MIROVA_VIIRS_ data analysis, we identify “Cycle 1” between 5 April 2013 and 29 October 2015, “Cycle 2” between 30 October 2015 and 22 November 2018, and “Cycle 3” between 23 November 2018 and 31 December 2020 (still ongoing). The highest VRP values were recorded during Cycle 2, after a phreatic eruption occurred on 30 October 2015 [63], and produced VRP values between 13 MW and 16 MW (see Figure 4 for details). Notably, the time gap between the thermal onset of MIROVA_VIIRS_ and MIROVA_MODIS_ (i.e., a sharp increase of VRP) characterizing each cycle was within 24 h, with a difference in the VRP value reached in the subsequent peak phases of less than 2 MW (Figure 4). This outlines that the three datasets provided consistent indications of abrupt changes in volcanic activity in both the timing (±24 h) and magnitude (±6%) of thermal emissions. However, it can be noted that the monthly-long phases preceding the climax of the three recognized cycles are characterized by few FIRMS alerts, while MIROVA_VIIRS_ detections outline the persistence of a very-low volcano-related thermal activity (Figure 4).

To exclude the possibility that these weak anomalies were false, we evaluated their accuracy through a visual inspection of all the alerts detected during 2012–2013 and we verified that they were always located inside the Lascar’s crater. The presence of a nearly-continuous low-level thermal activity during inter-eruptive periods was already retrieved and validated by previous analysis with other sensors (e.g., ASTER analysis; [3]).

The statistical analysis of the VRP dataset (log-transformed) may be useful to distinguish different thermal regimes and highlight threshold values separating different types of activity [96,97,98]. For long-term monitoring applications, it is therefore important that the datasets are complementary also in this aspect and that they allow the same thresholds to be calculated based on their statistical distributions.

**Figure 5:**
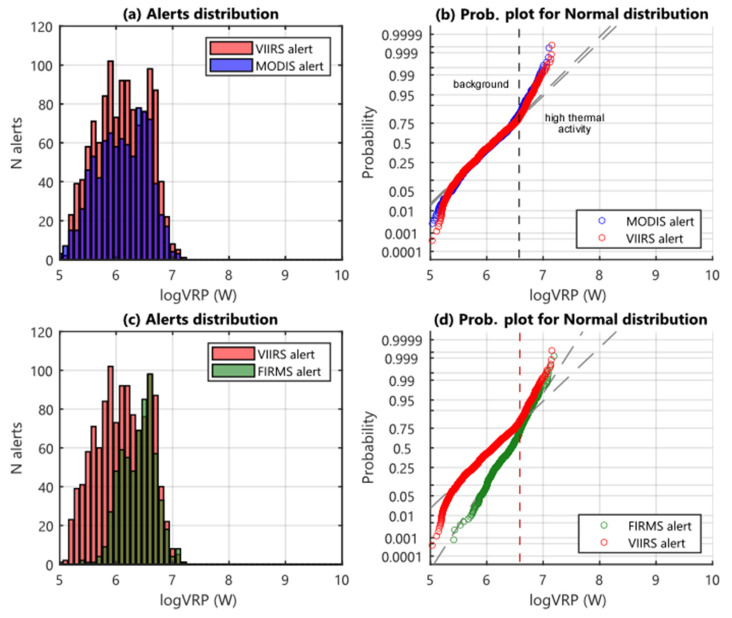
Histograms (**a**,**c**) and probability plots (**b**,**d**) for Láscar datasets. (**a**,**c**) Histograms display data distribution related to VRP (and FRP) in logarithmic scale (0.1 MW–1 GW), with a range between the classes of 0.1 bins; blue bars represent the distribution of MIROVA_MODIS_ data, the red ones represent MIROVA_VIIRS_ data, and green bars represent FIRMS_VIIRS_ data. (**b**,**d**) Probability plots for normal distribution of MIROVA_VIIRS_ (red), MIROVA_MODIS_ (blue), FIRMS_VIIRS_ (green) datasets; the dashed grey lines represent the reference lines of the theoretical distributions, and the black dashed line in (**b**) corresponds to the threshold between the background fumarolic activity and the higher thermal activity characterizing the eruptive phases. In (**d**) the red dashed line represents the slope change connected to the passage between the regimes of background and high thermal activity, detected only by the MIROVA_VIIRS_ algorithm. The probability plot of FIRMS data doesn’t show any significant changes, due to the low sensitivity of the algorithm on detecting volcano-derived low thermal anomalies.

The frequency distributions of MIROVA-derived VRP for Láscar are very similar for both VIIRS and MODIS, with data showing a range of VRP spanning from 0.1 to about 20 MW (Figure 5a). In general, there is a greater number of MIROVA_VIIRS_ alerts in each class in agreement with the greater number of VIIRS passages compared to MODIS (Table 2). In the probability plots (Figure 5b) the trends defined by these two datasets are also very similar both defining the presence of two different regimes separated by a threshold value of ~5 MW (logVRP ~6.7 in Figure 5a). We ascribe this threshold as separating two types of activity characterizing Láscar: (1) the near-continuous degassing activity (VRP < 5 MW) characterizing inter-eruptive periods, and (2) the main eruptive phases (VRP > 5 MW) associated with a moderate explosive activity eventually accompanied by episodes of dome extrusion or collapse. 

The presence of these two regimes, on the other hand, is not detectable from the FIRMS_VIIRS_ dataset, neither from the data distribution nor from the associated probability plot (Figure 5c,d). Indeed, there is a clear lack of FIRMS detection below 5 MW (logVRP ~6.7 in Figure 5c) suggesting that this algorithm is not able to fully detect the weak thermal anomalies inside the crater of Láscar (associated with high-temperature degassing activity). This result emerges clearly in the probability plot (Figure 5d) where there is a clear underestimation of FIRMS_VIIRS_ measurements for logVRP values <6.7, which does not allow to separate the two distinct thermal regimes outlined by MIROVA_VIIRS_ datasets.

**Figure 6:**
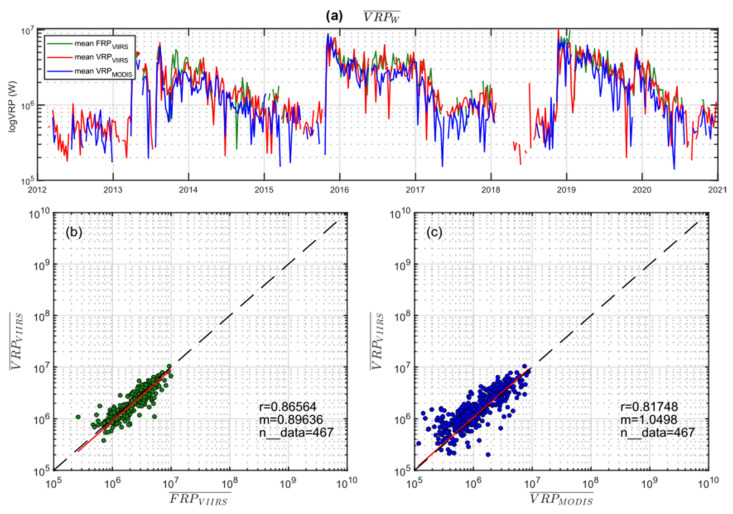
(**a**) Stacked time series of VRPw (weekly mean) retrieved for MIROVA_VIIRS_ (red), MIROVA_MODIS_ (blue), and FIRMS_VIIRS_ (green) at Láscar volcano in logarithmic scale. (**b**,**c**) Linear regression analyses between the weekly means of VRP_VIIRS_ and FRP_VIIRS_ (green), and VRP_VIIRS_ and VRP_MODIS_ (blue) at Láscar, respectively. The best-fit coefficient (m) close to 1 and the Pearson correlation coefficient r equal to 0.87 and 0.82, respectively, indicate an excellent correlation between the time series retrieved by MIROVA and FIRMS algorithms.

To better correlate the three time series, we calculated for each sensor the average values of VRP on a weekly scale (VRPw), as shown in Figure 6a, on a logarithmic scale. The three weekly datasets have been correlated by calculating the best-fit linear coefficient (m) and the Pearson correlation coefficient (r). From these analyses, it results that the MIROVA_VIIRS_ vs. MIROVA_MODIS_ and MIROVA_VIIRS_ vs. FIRMS_VIIRS_ are characterized by coefficient r equal to ~0.82 and ~0.87, respectively (Figure 6b,c). Data are slightly dispersed around the 1:1 ratio line, with a linear best-fit coefficient m of ~0.90 for MIROVA_VIIRS_ vs. FIRMS_VIIRS_ correlation, suggesting that MIROVA-derived weekly mean are slightly lower than FIRMS-derived FRP (Figure 6b). On the contrary, the coefficient m = 1.05 found for MIROVA-derived VRP (VIIRS vs. MODIS) indicates that the weekly mean for VIIRS is slightly higher than those obtained with MODIS (Figure 6c).

### 4.2. Erta Ale

**Figure 7:**
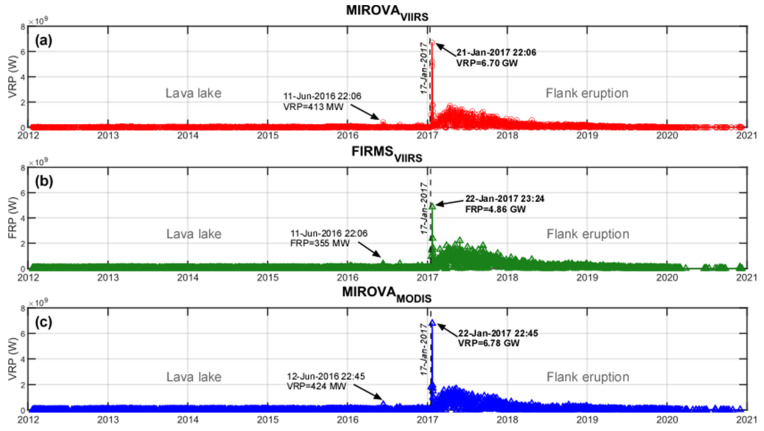
Time series of Erta Ale volcano. The panels show VRP calculated respectively from (**a**) MIROVA_VIIRS_ (red circles), (**b**) FIRMS_VIIRS_ (green triangles), and (**c**) MIROVA_MODIS_ (blue triangles). For both MIROVA and FIRMS, the peak in VRP and FRP reported on the time series with bold characters occurs after the onset of the flank eruption. The left dates on the time series correspond to the overflow that occurred in June 2016. Black dashed lines separate the two phases of Erta Ale’s activity.

The long-lasting activity of the Erta Ale is well represented by the continuous alerts detected by both sensors (Figure 7), corresponding to 2711, 1868 and 2816 for MIROVA_VIIRS_, MIROVA_MODIS,_ and FIRMS_VIIRS_, respectively (see Table 2). For this volcano, the MIROVA_VIIRS_ has therefore detected ~45% more alerts than the MIROVA_MODIS_ and ~3% fewer than the FIRMS_VIIRS_. Taking into consideration the number of satellite overpasses, the three systems had alert frequencies (f%) equal to ca. 70%, for MIROVA_VIIRS_, 63% MIROVA_MODIS_, and 72% for FIRMS_VIIRS_. 

According to the eruptive activity that occurred at Erta Ale during the analyzed period, the dataset may be subdivided into two main phases (Figure 7) characterized by: (1) lava lake activity (between 19 January 2012 and 16 January 2017) and (2) lava flow activity (between 17 January 2017 and 31 December 2020). The persistent thermal emission recorded until 2017 (fluctuating around 20 MW) is particularly stable within all datasets and likely related to the presence of the convective lava lake inside the southern pit crater. Few “spikes” are visible in this time interval (detected by both sensors and algorithms; Figure 7) probably outlining the occurrence of minor overflows from the lava lake(s). Successively, the sudden increase of VRP (occurred on 17 January 2017, marks the beginning of the lateral fissure eruption [77,81] and was recorded by the sudden increase of VRP by all the three systems (dashed line in Figure 7). The propagation of the lava flow in the following days led the VRP to increase rapidly, reaching peak values of 6.7 GW (MIROVA_VIIRS_), 6.8 GW (MIROVA_MODIS_), and 4.9 GW (FIRMS_VIIRS_) by 21–22 January (Table 2 and Figure 7). 

**Figure 8:**
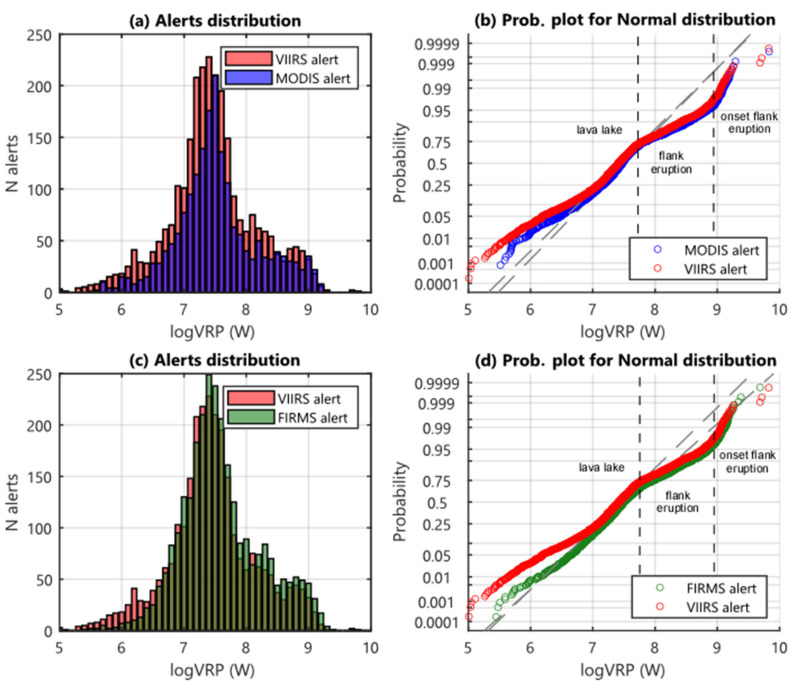
Histograms (**a**,**c**) and probability plot (**b**,**d**) for Erta Ale datasets. (**a**,**c**) Histograms display data distribution related to VRP in logarithmic scale (0.1 MW–1 GW), with a range between the classes of 0.1; blue bars represent the distribution of MODIS data, the red ones represent MIROVA_VIIRS_ data, and green bars represent FIRMS_VIIRS_ data. (**b**,**d**) Probability plots for normal distribution of MIROVA_VIIRS_ (red), MIROVA_MODIS_ (blue), FIRMS_VIIRS_ (green) datasets; the dashed grey lines represent the reference lines of the theoretical distributions. The main changes of the slope are identified with black dashed lines and correspond to the different regimes of activity that characterize Erta Ale’s last decade.

As shown in Figure 8a,c, Erta Ale’s data are characterized by very similar distributions with the highest number of detections occurring between 25 and 32 MW, for all systems (logVRP equal to 7.4–7.5 in Figure 8c). As for Láscar, our data suggest that VIIRS detected a higher number of anomalies than MODIS (+7%; Figure 8a) likely due to the higher number of overpasses (Table 2). On the other hand, the distributions of data from MIROVA_VIIRS_ and FIRMS_VIIRS_ are also very similar (Figure 8b). However, for logVRP < 6.7 MIROVA algorithm seems to be more sensitive, as confirmed by the very low number of FIRMS-derived detections below the 1 MW (logVRP = 6 in Figure 8c). 

All the three datasets outline that about 70% of detections are lower than 50 MW (logVRP equal to ~7.7; Figure 8b,d), a thermal regime that we ascribe to the typical lava lake activity characterizing Erta Ale volcano until early 2017 (Figure 7). Two other regimes emerge in all the analyzed datasets and are separated by a threshold of 1 GW (logVRP = 9 in Figure 8b,d). These two regimes are likely associated with the bulk effusive activity (50 MW < VRP < 1 GW; 22% of the data; Figure 8) and with the peaking phases of the effusive activity (VRP > 1 GW; less than 4% of the data). As previously cited, these different phases are well recorded by both sensors and algorithms, also showing a time homogeneity observable in the time series. 

**Figure 9:**
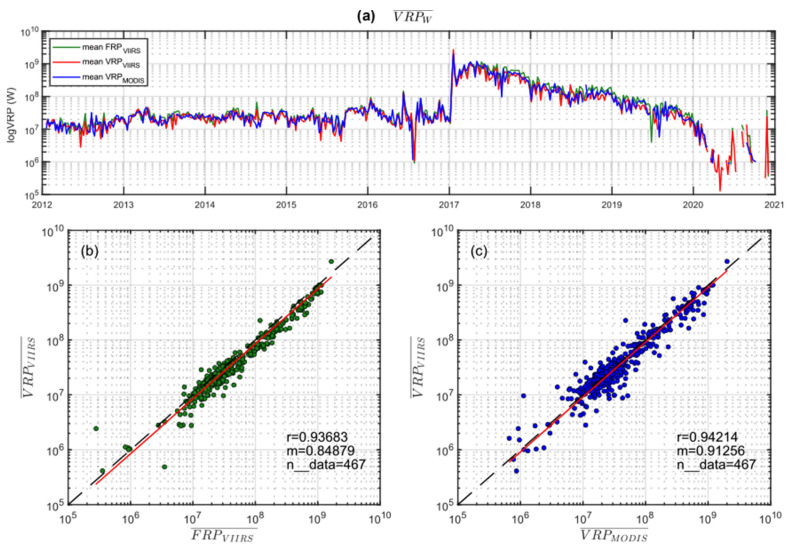
(**a**) Stacked time series of VRPw retrieved for MIROVA_VIIRS_ (red), MIROVA_MODIS_ (blue), and FIRMS_VIIRS_ (green) at Erta Ale volcano in logarithmic scale. (**b**,**c**) Linear regression analyses between VRP_VIIRS_ and FRP_VIIRS_-derived VRPw (green), and VRP_VIIRS_ and VRP_MODIS_-derived VRPw (blue) at Erta Ale, respectively. The best-fit coefficients m close to 0.9 and the Pearson correlation coefficients r ~0.94 indicate an excellent correlation between the time series retrieved by MIROVA and FIRMS algorithms.

The weekly averaged dataset (Figure 9a) shows comparable and almost stackable trends, within the full ranges of VRP/FRP values, spanning over 4 orders of magnitude (from less than 1 MW to more than 1 GW). The linear regression analysis (Figure 9b,c) confirms the strong correlation between the three datasets (r~0.94 for both MIROVA_VIIRS_ vs. MIROVA_MODIS_ and MIROVA_VIIRS_ vs. FIRMS_VIIRS_) with best-fit coefficients equal to m=0.85 (for MIROVA_VIIRS_ vs. FIRMS_VIIRS_) and m=0.91 (for MIROVA_VIIRS_ vs. MIROVA_MODIS_). Accordingly, also for Erta Ale, there is an excellent correspondence between the three datasets, with a tendency of MIROVA_VIIRS_ to produce weekly values slightly lower than the other systems.

### 4.3. Cumulative Radiant Energy (VRE) Calculation via VRP Datasets

**Figure 10:**
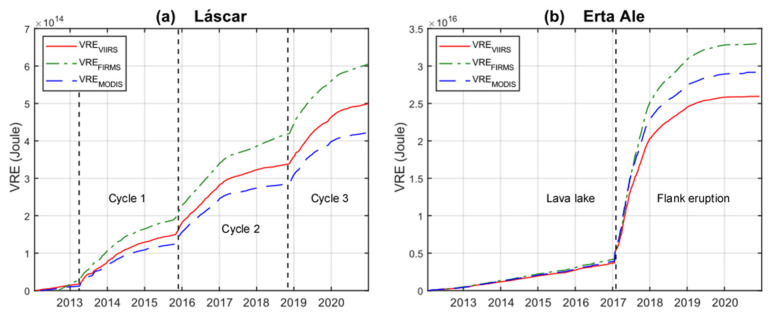
Cumulative Volcanic Radiative Energy (VRE) calculated from VRP (and FRP) by trapezoidal rule for integration: the red line represents VRE_VIIRS_, the blue dashed line VRE_MODIS_ and the green dashed line VRE_FIRMS_. (**a**) Láscar; (**b**) Erta Ale. Dashed black lines are the limits of the three cycles of Láscar and the two phases of Erta Ale’s eruptive history considered in this paper.

A further element of comparison between the three datasets is provided by the cumulative curves of the radiated volcanic energy (VRE in Figure 10). The VRE is a fundamental parameter since it allows to compare eruptions and during the effusive eruptions it is directly correlated to the volume of erupted lava [30]. It is therefore useful to evaluate if the three systems provide comparable VRE values over time.

VRE was calculated by using the trapezoidal integration of VRP/FRP time series, whose cumulative curves are shown in Figure 10. In Table 3 we reported the VRE values for the two analyzed volcanoes, also subdivided based on the eruptive phases identified above. At Láscar we observe that the VRE_VIIRS_ is about 18% higher than VRE_MODIS_ and 21% lower than VRE_FIRMS_. These differences are somehow coherent with the correlation coefficient (m) characterizing the weekly values (as shown in Figure 6) but are possibly biased also by the number and distribution of VRP values in each dataset. Indeed, in the case of FIRMS, the lack of many low-intensity detections (Figure 5c) produces an integrated VRE curve higher than in the other datasets. On the other hand, no remarkable variations are observed in the ratio of the VRE throughout the eruption which remains roughly constant during all three cycles (Table 3). 

Similarly, at Erta Ale we found that the dataset producing the higher VRE is FIRMS_VIIRS,_ followed by MIROVA_MODIS_ and MIROVA_VIIRS_ (Figure 10b). The relationship between the energies is partially influenced by the coefficient m found for Erta Ale (Figure 9), which is indicative of VRP_VIIRS_ values slightly lower than VRP_MODIS_ and FRP_VIIRS_. However, we found that most of the discrepancy between the cumulative curves is essentially produced during the effusive phase (Table 3 and Figure 10b). In fact, during the effusive eruption, there was a tendency for FIRMS_VIIRS_ to measure energies slightly higher than the MIROVA_VIIRS_ (Figure 9a). This variation suggests that the type of activity (i.e., lava lake vs. lava flow) influenced the correlation between the analyzed datasets, even if the bulk of three VREs remains within a difference of ±20%.

## 5. Toward Global Volcano Thermal Monitoring Using VIIRS

The results presented above clearly highlight as the VIIRS and MODIS imagery, processed by the MIROVA system, produce consistent results in terms of VRP values, statistical distribution, the timing of major changes in volcanic activity, and cumulative thermal energy (VRE). These analyses were carried out on two volcanoes (Láscar and Erta Ale) specifically chosen to compare the nighttime long datasets also with the data provided by the FIRMS system, which is based on a different algorithm. However, to test the exportability of the MIROVA_VIIRS_ algorithm for a near-real-time and near-global application (as currently made for MODIS), we have to consider the multitude of environmental contexts in which volcanoes are located, as well as the full potential of the VIIRS suite, now constituted by two sensors each one providing nighttime and daytime images.

For this purpose, we analyzed a one-year-long dataset (all daytime and nighttime images between January–December 2021) acquired over 9 persistently active volcanoes (Figure 1) by the pair of VIIRS sensors currently onboard Suomi-NPP and NOAA-20 satellites, respectively. The resulting VRP time series (Supplementary material) are then compared with those provided by the operational MIROVA_MODIS_ system that includes both night and daytime imagery from Terra and Aqua satellites [7]. 

**Figure 11:**
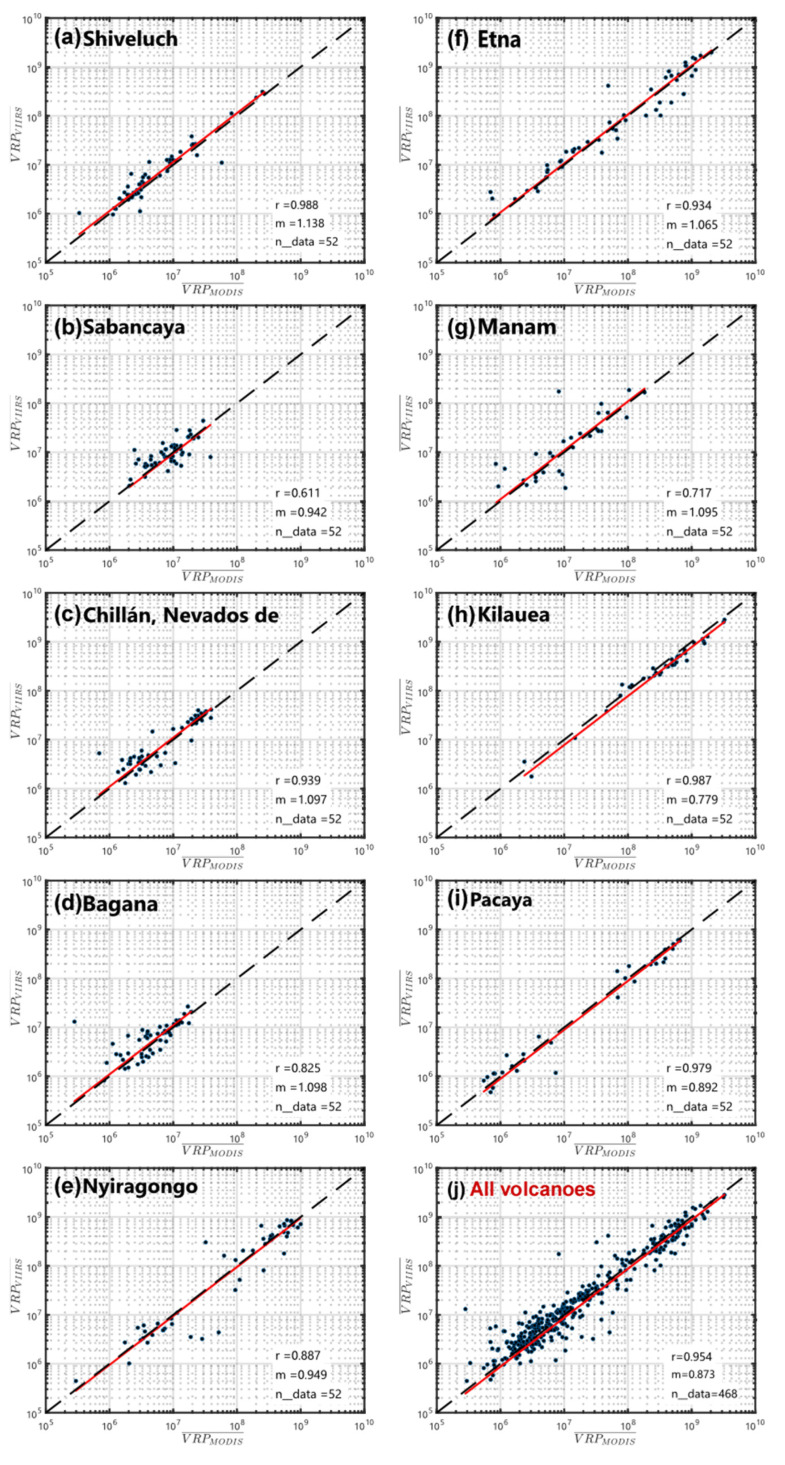
Linear regressions VIIRS and MODIS-derived VRPw (weekly mean) of subaerial volcanoes: (**a**) Shiveluch, Russia; (**b**) Sabancaya, Peru; (**c**) Nevados de Chillán, Chile; (**d**) Bagana, Papua New Guinea; (**e**) Nyiragongo, D.R. Congo; (**f**) Etna, Italy; (**g**) Manam, Papua New Guinea; (**h**) Kilauea, Hawaii; (**i**) Pacaya, Guatemala. (**j**) Whole dataset (9 volcanoes). Red lines represent the best-fit linear correlation whose coefficients m and r are reported in each graphic. Black dashed lines are the 1:1 ratio.

The chosen volcanic targets (Table 4 and Figure 11) show thermal emissions ranging from about 1 MW to more than 1 GW and embed a variety of volcanic activity that includes: silicic lava domes (Shiveluch, Sabancaya, Nevados de Chillan), andesitic lava domes (Bagana), lava lakes (Nyiragongo), open vent (Etna, Manam), and mafic lava flow (Kilauea, Pacaya). Some of these volcanoes have experienced important eruptive phases in the considered period (see Supplementary Material), such as Etna [99,100], Nyiragongo volcano [101], and Kilauea [102]. However, in addition to their recent activity, these volcanoes have been chosen because they are representative of a wide range of environmental and meteorological conditions spanning from ice-covered high-latitude volcanoes (e.g., Sheveluch) to tropical islands (e.g., Bagana, Kilauea). We, therefore, focus on evaluating the performance of the MIROVA_VIIRS_ algorithm at each volcano by analyzing: (i) the number of alerts, (ii) the correlations coefficient between weekly VRP values, (iii) the nighttime vs. daytime performance.

*The number of alerts*: Overall, our analysis reveals that the VIIRS sensors provide about 30% more overpasses than the MODIS (17487 vs. 13416; Table 4). This is due to the larger swath of VIIRS (Table 1) that allows, in some cases, imaging a target volcano even on multiple adjacent orbits. Although this high number of passages is characterized by frequent acquisitions with very high satellite zeniths (many VIIRS passages are characterized by angles greater than 55°, the limit of MODIS), the total number of VIIRS alerts is on average 62% more than MODIS (6293 vs. 3883; Table 4), indicating an increased efficiency in detecting hot-spots. It should be emphasized that the increase of VIIRS detections is inversely proportional to the average VRP (Table 4), and growth from 30–50% at highly radiating volcanoes (such as Kilauea, Etna, Nyiragongo, and Pacaya), to more than 100% on volcanoes characterized by low thermal flux (e.g., Manam, Bagana, Sabancaya). This makes the VIIRS more effective than MODIS in monitoring background thermal emissions at persistently active volcanoes (Supplementary Material), but also more suitable for detecting thermal precursors at restless volcanoes [7].

We suggest that the higher efficiency of VIIRS, with respect to MODIS, is related to a combination of improved instrument features such as the higher spatial resolution, the lower noise equivalent temperature (NedT), and the already-mentioned aggregation function that allows keeping the spatial resolution below 1.5 km also in extreme viewing conditions (see Table 1). All these features increase the possibility of detecting small sub-pixel hot-spots and ensure a better location of the alerted pixels, too (Figure 2 and Figure 3). 

*Correlation Coefficient*: The correlation found for every single volcano as well as the total dataset (all volcanoes together) is shown in Figure 11. The Pearson coefficient (r) confirms the good correlation between VIIRS- and MODIS-derived VRP, with values ranging from 0.62 (Sabancaya; Figure 11b) to 0.99 (Shiveluch; Figure 11a). Similarly, the best-fitting coefficient (m) is found to vary between 0.78 (Kilauea; Figure 11h) and 1.14 (Shiveluch; Figure 11a), possibly reflecting the influence of variable environmental, meteorological, and volcanological conditions characterizing such a relationship at each distinct volcano. Taken as a whole the bulk correlation (Figure 11j) give a Pearson coefficient r = 0.95 and a regression coefficient m = 0.87, delineating an overall discrepancy between VRP_VIIRS_ and VRP_MODIS_ of ~13%. These results underline the excellent correlation between the two datasets although it is also evident that local factors can play a role in determining a case-by-case variance in the estimate of weekly VRP (typically less than 20%). 

As we have discussed previously, the local correlation coefficients are likely conditioned by the number and distribution of the data, by the type of volcanic activity (stable or fluctuating) as well as by the environmental conditions that may condition the performance of the underlying algorithms. 

*Nighttime vs daytime data*: The comparative analysis confirms that the performance of MIROVA algorithm decreases when applied to the daytime data. In particular, we observed a reduction in the frequency of alerts (f% = N_alert_ / N_pass_) from 51% (nighttime) to 20% (daytime) for the VIIRS, compared to a reduction from 43% (nighttime) to 16% (daytime) measured with the MODIS (Table 4). As described in [26], this strong reduction is due to the higher thresholds of the daytime algorithm specifically settled to avoid false alerts due to the contribution of solar heating and reflection. The reduction is particularly evident in volcanoes characterized by a general low heat emission (e.g., Manam, Bagana, Sabancaya) for which thermal anomalies are detected mostly at night. 

## 6. Conclusions

In this paper, we presented VRP time series, and cumulative VRE data obtained on two selected target volcanoes, by applying an adapted version of the MIROVA algorithm to VIIRS imagery. We thus analyzed the efficiency of the MIROVA_VIIRS_ algorithm by comparing the obtained time series with those provided by the operative MIROVA_MODIS_ algorithm [55] as well as by the FRP time series provided by the FIRMS system [48]. The comparative analysis at these two specific volcanoes allowed us to conclude that:

- by using the MIROVA algorithm, the VIIRS sensor detects ~40% more alerts than MODIS (on average). This difference is likely due to the greater number of VIIRS overpasses, compared to MODIS (+29% on average), but also to a better quality of the VIIRS images which make the hot-spot detection more efficient (e.g., better spatial resolution, better pixel aggregation at high satellite zenith angle, less NEdT);
- the two MIROVA-derived datasets are highly correlated each other (r = 0.81–0.94 for Láscar and Erta Ale, respectively) and show a best-fit linear coefficient (m = VRP_VIIRS_/VRP_MODIS_) around the 1:1 ratio, ranging from 0.91 (Erta Ale) to 1.05 (Láscar); the two datasets are also comparable in terms of VRP distributions (modal value ±5%), the timing of major events (within 24 h), and cumulative radiant energy (±18%);
- the comparison of the VIIRS data processed with FIRMS algorithms instead reveals a better ability of the MIROVA algorithm to detect small thermal anomalies (<10 MW). At volcanoes characterized by low-amplitude thermal anomalies (such as Láscar), this difference translates into an increase of up to 95% of the number of alerts detected by MIROVA_VIIRS_ compared to the FIRMS_VIIRS_. On the other hand, the two algorithms, appear to be equally efficient on volcanoes characterized by a more intense thermal activity (e.g., Erta Ale);
- regardless of the frequency of detections the VRP time series retrieved from MIROVA_VIIRS_ and FIRMS_VIIRS_ are highly correlated (r = 0.87 to 0.94) and show a best-fit linear regression coefficient (m = VRP_VIIRS_/FRP_FIRMS_) equal to 0.90 (Láscar) and 0.84 (Erta Ale);

Finally, to test the global application of the MIROVA_VIIRS_ algorithm, we analyzed one year of nighttime and daytime data acquired by the two pairs of VIIRS and MODIS sensors. This analysis has been performed at 9 volcanoes located in different environments and characterized by very different volcanic activity. The bulk linear correlation coefficient suggests a best-fit value (m = VRP_VIIRS_/VRP_MODIS_) equal to 0.87 (r = 0.95), with single volcanoes coefficients spanning from 0.78 to 1.14 (Table 4). Based on this analysis we may conclude that the VIIRS detects from 32 to 108% more alerts than MODIS with an average of + 62% on the entire dataset. This percentage varies from volcano to volcano and, as previously mentioned, it is likely to be attributed to the improved features of the VIIRS instrument that allow detecting smaller thermal anomalies. 

The above points confirm that the VIIRS instrument is fully consistent with MODIS and suitable for replacing and improving the latter in monitoring high-temperature volcanic heat emissions for the next decades. Moreover, our results suggest that the datasets processed by the MIROVA and FIRMS systems are comparable and provide consistent estimates on the radiant power sourced by volcanic eruptions. This cross-validation strengthens, even more, the value of the VRP which therefore constitutes a robust, multi-platform, multi-algorithm, indicator of volcanic thermal activity. On the other hand, the MIROVA algorithm, and in particular the MIROVA_VIIRS_ version, turns out to be more efficient in detecting low-intensity thermal anomalies (<10 MW). This greater efficiency translates into a better ability to discriminate between background and anomaly values (cf. Figure 6) as well as to detect small signs of activity, otherwise undetected by the FIRMS. In terms of volcanic surveillance, this difference can be very significant at restless volcanoes as the first appearance of thermal activity can be detected days, weeks, or even months in advance compared to MODIS [7]. In this view, the application of the MIROVA algorithm to VIIRS data at 375 m resolution has all the potential to improve the detection capability of hot-spots during volcanic unrest phases as recently proved by [103].

Given the imminent end of the EOS missions, the future JPSS missions (the next scheduled for 2022, [104]) assume a key role. They will both fill the lack of MODIS data and potentially improve the monitoring capabilities with a higher number of daily acquisitions. On the other hand, our results show that there is margin for improvement in thermal remote sensing of volcanic activity, especially if future space missions, expressly dedicated to volcanic monitoring [46], will provide mid-infrared data with high spatial and high temporal resolutions in continuity with the VRP time series inherited from MODIS and VIIRS.

## Figures and Tables

**Table 1:** Main characteristics and spectral bands of VIIRS and MODIS.

	VIIRS (S-NPP/N20)	MODIS (TERRA/AQUA)
*Orbit altitude (km)*	824	705
*Equator crossing time*	13:30 LT/12:40 LT	10:30 LT/13:30 LT
*Swath (km)*	3060	2330
*Pixel resolution at nadir (km)*	0.75–0.375	1
*Pixel resolution at the edge (km)*	1.5–0.75	4
*Spectral coverage* *of* *thermal bands (* *μ* *m)*	3.550–12.488	3.660–14.385
*Number of thermal bands*	7	14
*ID MIR Band(s)*	M-13	21 22
*Spectral range (μm)*	3.973–4.128	3.929–3.989 3.940–4.001
*T_MAX_ (SNR-NEdT on orbit)*	634 K (0. 04)	500 K (0.183) 331 K (0.019)
*ID TIR* *Band(s)*	M-15	31
*Spectral range (μm)*	10.263–11.263	10.780–11.280
*T_MAX_ (SNR-NEdT on orbit)*	343 K (0.03)	400 K (0.017)

**Table 2:** Comparison between the datasets MIROVA_VIIRS_, MIROVA_MODIS_ and FIRMS_VIIRS_ for Láscar and Erta Ale volcanoes. The summary includes the number of overpasses (N_pass_), the number of alerts (N_alerts_) including the frequency (f%) of alerts (N_alert_/N_pass_), the N_alert_ ratio (ratio between the number of alerts detected by the different algorithms) as well as the mean and maximum VRP/FRP obtained for each dataset.

Volcano	#	Láscar	Erta Ale
N_pass_	MIROVA_VIIRS_	4049	3885
	MIROVA_MODIS_	3164	2977
	FIRMS_VIIRS_	4049	3885
N_alerts_ (f%)	MIROVA_VIIRS_	1221 (30%)	2711 (70%)
	MIROVA_MODIS_	875 (28%)	1868 (63%)
	FIRMS_VIIRS_	627 (15%)	2816 (72%)
N_alert_ ratio	MIROVA_VIIRS_/ MIROVA_MODIS_	140%	145%
	MIROVA_VIIRS_/FIRMS_VIIRS_	195%	96%
Mean VRP (MW)	MIROVA_VIIRS_	1.78	92.6
	MIROVA_MODIS_	1.53	104.5
	FIRMS_VIIRS_	2.31	117.9
Max VRP (MW)	MIROVA_VIIRS_	14.25	6700.7
	MIROVA_MODIS_	12.66	6781.1
	FIRMS_VIIRS_	15.55	4863.9

**Table 3:** Table displaying cumulative total VRE for every Láscar cycle and Erta Ale phase. In grey cells, there are the same values calculated for the period analyzed in this work (2012–2020).

		VRE (J)		
		MIROVA_VIIRS_	FIRMS_VIIRS_	MIROVA_MODIS_
*Láscar*	5 Apr 2013–30 Oct 2015	$1.32\times 10^{14}$	$1.59\times 10^{14}$	$1.11\times 10^{14}$
	31 Oct 2015–23 Nov 2018	$1.89\times 10^{14}$	$2.27\times 10^{14}$	$1.61\times 10^{14}$
	24 Nov 2018–31 Dec 2020	$1.57\times 10^{14}$	$1.82\times 10^{14}$	$1.33\times 10^{14}$
	2012–2020	$4.99\times 10^{14}$	$6.05\times 10^{14}$	$4.22\times 10^{14}$
*Erta Ale*	19 Jan 2012–16 Jan 2017	$3.69\times 10^{15}$	$4.19\times 10^{15}$	$3.91\times 10^{15}$
	17 Jan 2017–31 Dec 2020	$2.22\times 10^{16}$	$2.88\times 10^{16}$	$2.52\times 10^{16}$
	2012–2020	$2.59\times 10^{16}$	$3.30\times 10^{16}$	$2.92\times 10^{16}$

**Table 4:** Comparison between the 2021 datasets of VIIRS and MODIS for selected volcanoes. The summary includes the correlation coefficient shown in Figure 11, the number of overpasses (N_pass_), the number (N_alerts_), the frequency (f% = N_alert_/N_pass_) of alerts (subdivided into nighttime and daytime data), the ratio between VIIRS and MODIS detections, and the mean VRP obtained by averaging the weekly dataset showed in Figure 11 and Supplementary Material.

Volcano	Correlation Coefficient		N_pass_		N_alerts_ (f%)		N_alert_ ratio	f% (N_alert_/N_pass_) Nighttime		f% (N_alert_/N_pass)_ Daytime		Mean VRP (MW)	
	m	r	VIIRS	MODIS	VIIRS	MODIS	VIIRS/MODIS	VIIRS	MODIS	VIIRS	MODIS	VIIRS	MODIS
Shiveluch (Kamchatcka)	1.138	0.988	3264	2424	1098 (33.6%)	655 (27%)	167.6%	45.3%	45.3%	23.3%	23.3%	26.2	22.8
Sabancaya (Chile)	0.942	0.611	1684	1287	689 (40.9%)	374 (29.1%)	184.2%	69.5%	55.0%	13.7%	3.5%	11.1	10.3
Chillán, Nevados de (Chile)	1.097	0.939	2109	1617	970 (46%)	574 (35.5%)	169.0%	71.4%	57.5%	21.2%	14.1%	13.8	12.1
Bagana (Bouganville Island)	1.098	0.825	1664	1291	364 (21.9%)	175 (13.6%)	208.0%	40.1%	25.1%	3.4%	2.2%	7.4	6.2
Nyiragongo (DRC)	0.949	0.887	1576	1274	530 (33.6%)	368 (28.9%)	144.0%	53.3%	45.5%	14.0%	12.6%	254.4	247.9
Etna (Italy)	1.065	0.934	2095	1605	999 (47.7%)	646 (40.2%)	154.6%	68.9%	62.0%	26.8%	18.4%	339.9	314.0
Manam (Papua New Guinea)	1.095	0.717	1640	1275	317 (19.3%)	157 (12.3%)	201.9%	20.0%	14.6%	18.7%	10.1%	36.1	26.3
Kilauea (Hawaii)	0.779	0.987	1732	1324	742 (42.8%	559 (42.2%)	132.7%	47.5%	47.6%	38.2%	36.9%	527.7	685.2
Pacaya (Guatemala)	0.892	0.979	1723	1319	584 (33.9%)	375 (28.40)	155.7%	42.9%	35.3%	24.7%	21.6%	161.3	178.8
**Whole**	**0.873**	**0.954**	**17487**	**13416**	**6293 (36.0%)**	**3883 (28.9%)**	**162.1%**	**51.0%**	**43.1%**	**20.4%**	**15.9%**	**153.1**	**167.1**

## Data Availability

The data presented in this study are available on request from the corresponding author.

## Supplementary Materials

The following supporting information can be downloaded at: https://www.mdpi.com/article/10.3390/s22051713/s1.
