# Supplementary material for: The Transition from MODIS to VIIRS for Global Volcano Thermal Monitoring
Source: Sensors (Basel). 2022 Feb 22;22(5):1713. doi: 10.3390/s22051713 (PMC8914890; doi:10.3390/s22051713)
Supplement: Supplementary file 1 [file sensors-22-01713-s001.zip › sensors-1545034-supplementary.pdf]

sensors

MDPI

Supplementary Materials

In the following supplementary section, we present the datasets of the 9 volcanoes analyzed in Section 5. We plotted in Figures S1-S9 both VRP_{VIIRS} and VRP_{MODIS} one-year-long time series, and the weekly values (VRP_W; black lines) used for the correlation shown in Figure 11.

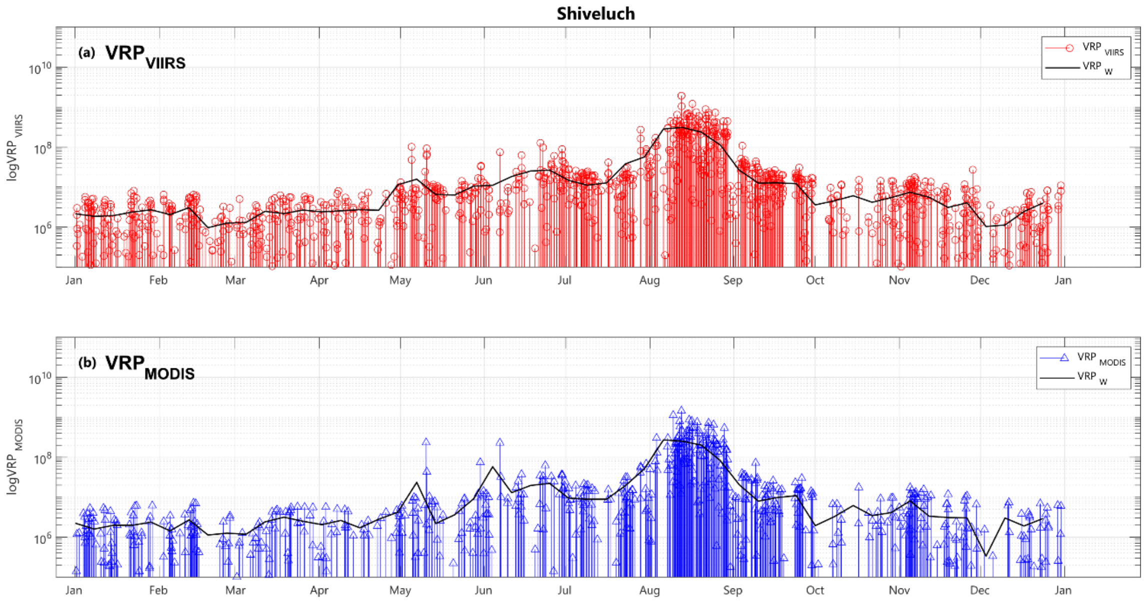

Figure S1. Time series of Shiveluch volcano. The panels show VRP calculated respectively from MIROVA-VIIRS ((a), red circles), MODIS ((b), blue triangles), in logarithmic scale related to the year 2021. The black line represents the weekly-mean VRP values used to evaluate the correlation in Section 5 (Figure 11a).

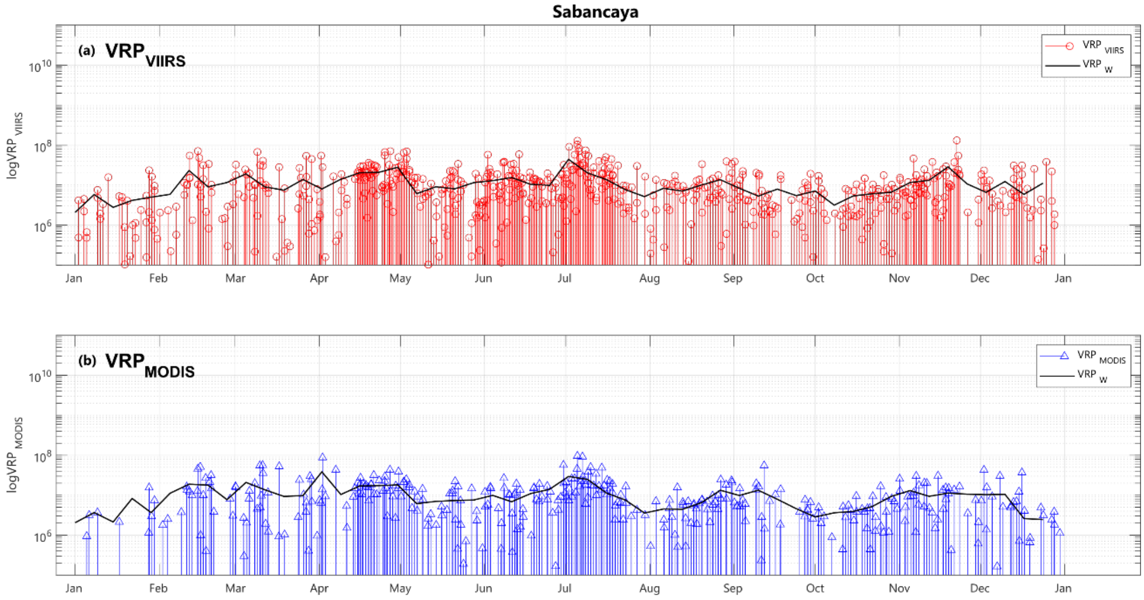

Figure S2. Time series of Sabancaya volcano. The panels show VRP calculated respectively from MIROVA-VIIRS ((a), red circles), MODIS ((b), blue triangles), in logarithmic scale related to the year 2021. The black line represents the weekly-mean VRP values used to evaluate the correlation in Section 5 (Figure 11b).

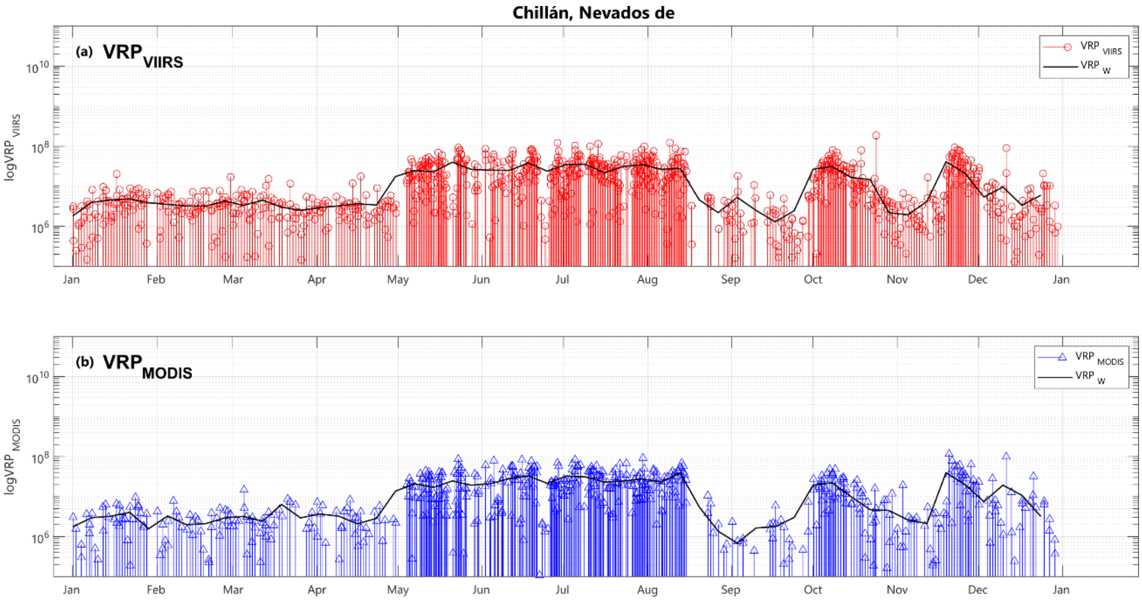

Figure S3. Time series of Nevados de Chillán volcano. The panels show VRP calculated respectively from MIROVA-VIIRS ((a), red circles), MODIS ((b), blue triangles), in logarithmic scale related to the year 2021. The black line represents the weekly-mean VRP values used to evaluate the correlation in Section 5 (Figure 11c).

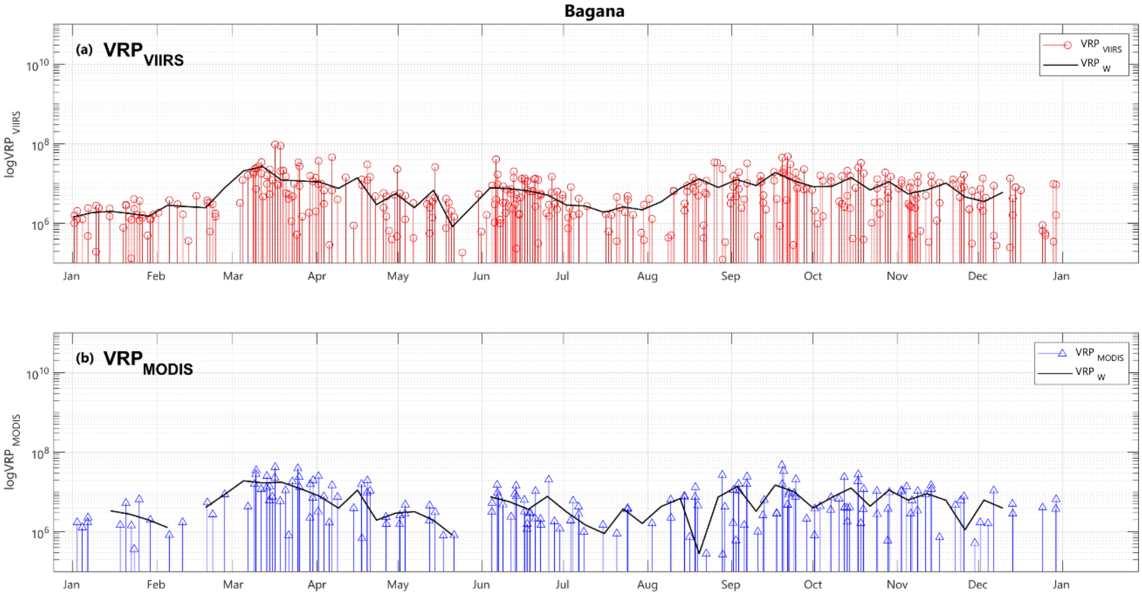

Figure S4. Time series of Bagana volcano. The panels show VRP calculated respectively from MIROVA-VIIRS ((a), red circles), MODIS ((b), blue triangles), in logarithmic scale related to the year 2021. The black line represents the weekly-mean VRP values used to evaluate the correlation in Section 5 (Figure 11d).

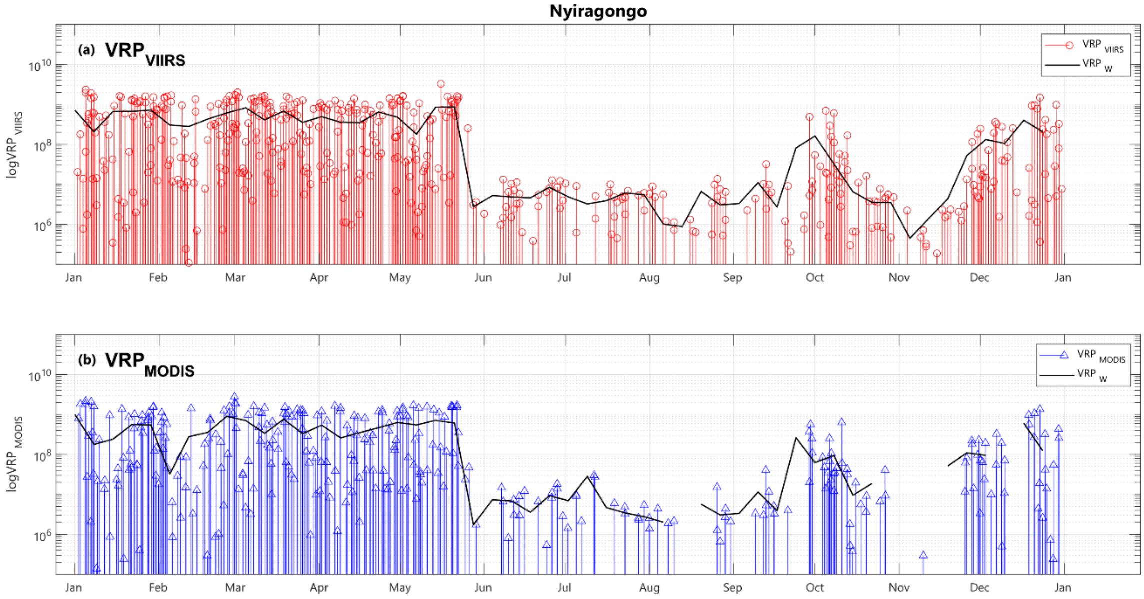

Figure S5. Time series of Nyiragongo volcano. The panels show VRP calculated respectively from MIROVA-VIIRS ((a), red circles), MODIS ((b), blue triangles), in logarithmic scale related to the year 2021. The black line represents the weekly-mean VRP values used to evaluate the correlation in Section 5 (Figure 11e).

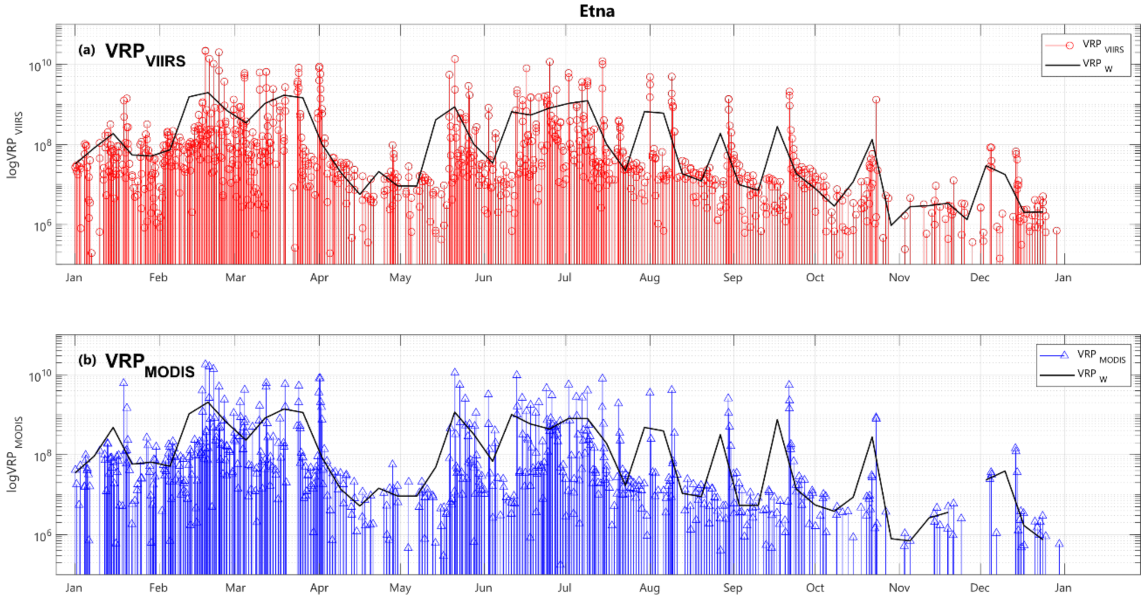

Figure S6. Time series of Etna volcano. The panels show VRP calculated respectively from MIROVA-VIIRS ((a), red circles), MODIS ((b), blue triangles), in logarithmic scale related to the year 2021. The black line represents the weekly-mean VRP values used to evaluate the correlation in Section 5 (Figure 11f).

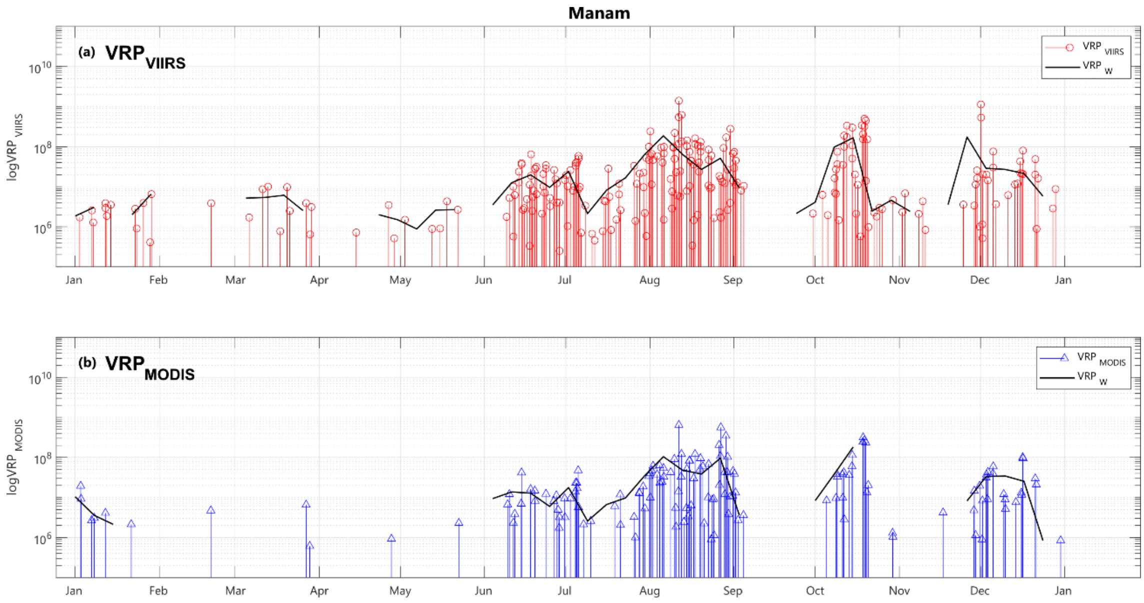

Figure S7. Time series of Manam volcano. The panels show VRP calculated respectively from MIROVA-VIIRS ((a), red circles), MODIS ((b), blue triangles), in logarithmic scale related to the year 2021. The black line represents the weekly-mean VRP values used to evaluate the correlation in Section 5 (Figure 11g).

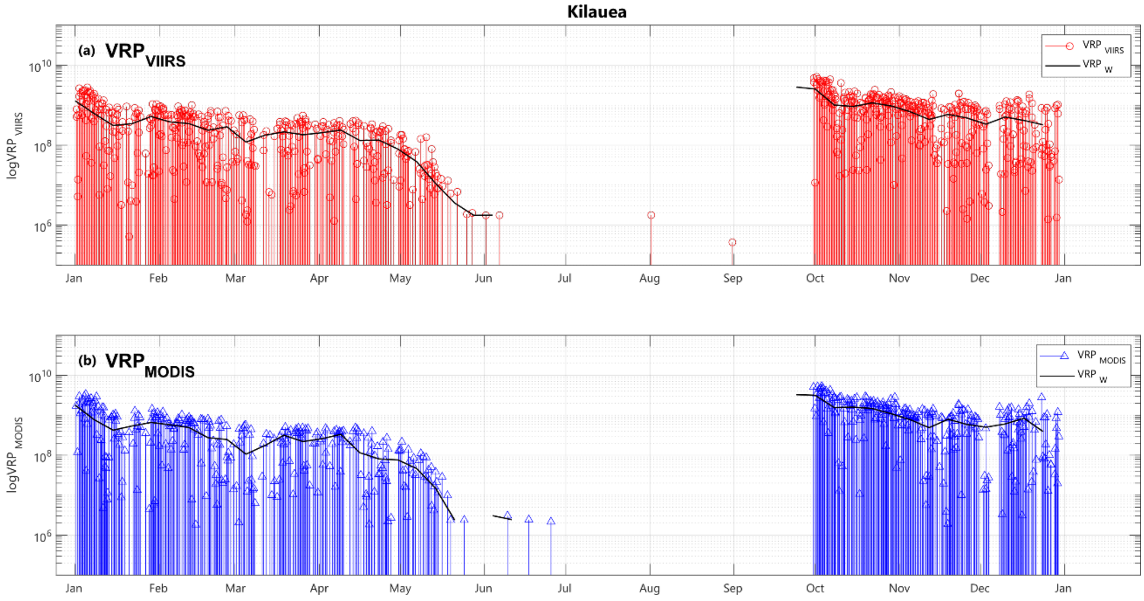

Figure S8. Time series of Kilauea volcano. The panels show VRP calculated respectively from MIROVA-VIIRS ((a), red circles), MODIS ((b), blue triangles), in logarithmic scale related to the year 2021. The black line represents the weekly-mean VRP values used to evaluate the correlation in Section 5 (Figure 11h).

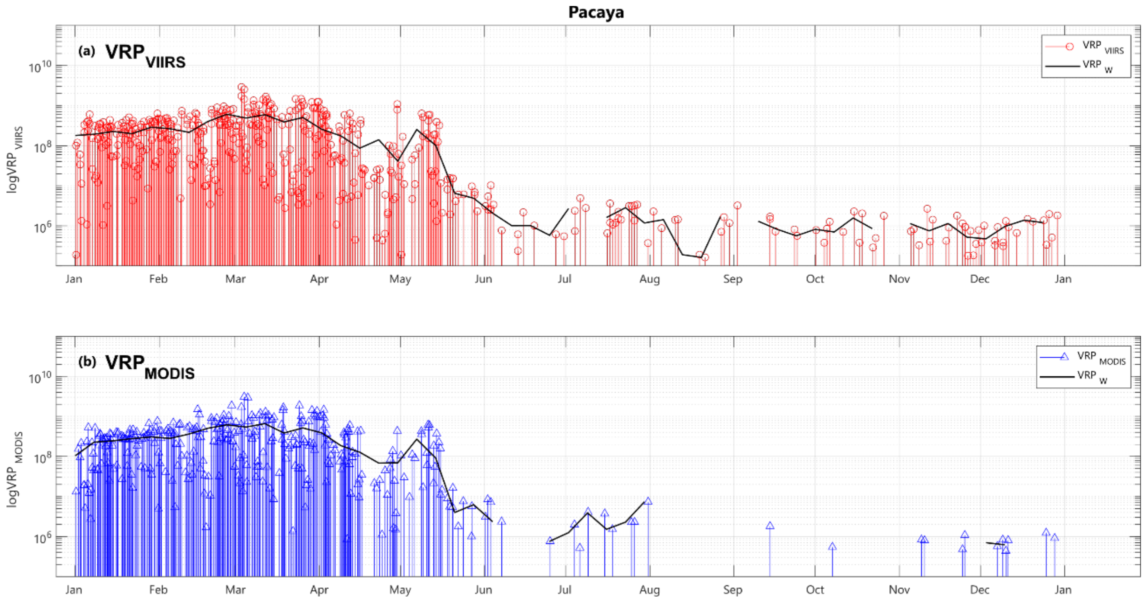

Figure S9. Time series of Pacaya volcano. The panels show VRP calculated respectively from MIROVA-VIIRS ((a), red circles), MODIS ((b), blue triangles), in logarithmic scale related to the year 2021. The black line represents the weekly-mean VRP values used to evaluate the correlation in Section 5 (Figure 11i).
